# Influence of Cryo-Processing and Post-SPD Annealing on Creep Behavior of CP Titanium

**DOI:** 10.3390/ma15051646

**Published:** 2022-02-22

**Authors:** Jiri Dvorak, Petr Kral, Andrey G. Kadomtsev, Vladimir I. Betekhtin, Maria V. Narykova, Marie Kvapilova, Vaclav Sklenicka

**Affiliations:** 1 Institute of Physics of Materials, Czech Academy of Sciences, Zizkova 22, 616 62 Brno, Czech Republic; pkral@ipm.cz (P.K.); kvapilova@ipm.cz (M.K.); sklen@ipm.cz (V.S.); 2 Ioffe Physical-Technical Institute, Russian Academy of Sciences, Politechnicheskaya 26, 194 021 St. Petersburg, Russia; andrej.kadomtsev@mail.ioffe.ru (A.G.K.); vladimir.betekhtin@mail.ioffe.ru (V.I.B.); maria.narykova@mail.ioffe.ru (M.V.N.)

**Keywords:** titanium, creep properties, severe plastic deformation, static annealing, cryo-processing

## Abstract

The commercial purity of VT1-0 titanium was processed by the rolling process and executed at elevated, room, and cryo-temperatures. These processings led to the formation of an ultrafine-grained microstructure, with the mean grain size at a nanometer level. Some of these materials were statically annealed at a temperature of 823 K for 1 h, which led to significant subgrains and grain coarsening. The constant load creep tests in tension were carried out in argon on all states of materials, at temperatures of 648–723 K and different ranges of applied stresses. From the value of the steady-state creep rate, the control creep mechanisms were determined. The microstructure analyses were carried out via SEM and TEM. It was found that titanium prepared at elevated and room temperatures have a higher creep strength than titanium prepared at cryo-temperatures. Furthermore, the post-SPD —annealing led to a significant decrease in the creep properties. The influence of the preparation temperature on the difference of the creep behavior were discussed and explained using the microstructure analyses of the tests’ samples.

## 1. Introduction

Titanium and its alloys are widely used both at room temperature and elevated temperatures. For this reason, they are often used for components in the automotive or aerospace industry. Ti base materials are mainly characterized by an excellent strength at a low density (high specific strength) and corrosion resistance. The strength of Ti at room temperature (RT) can be significantly improved by an application of the methods of severe plastic deformation (SPD), which reduce the grain size down to a submicroscopic scale [1,2]. However, the grain size depends on the value of the imposed strain during SPD. It is now established that a larger imposed strain results in a finer grain size. However, at extremely high strains, the saturation of the microstructure characteristics [3,4] occurs, and the grain size reaches almost the size of the subgrains. The grain size refinement by SPD seems to have certain limits. However, during SPD, it is possible to change not only the level of imposed strain but also the deformation temperature. 

The previous works showed that the reduction of the SPD processing temperature down to a cryogenic level could lead to the further refinement in grain size down to nanoscopic level [5,6,7]. However, the microstructure formed at low temperatures may possess a low-temperature microstructure stability, and the grain growth or recrystallization can occur even at room temperature [5,6,7,8,9]. The microstructure of materials such as Mg, Ag, or Al deformed at low-temperatures (below RT) are unstable even at RT after short-term storage [5,6,7]. It was also found that pure Al and Cu deformed at RT are not stable during long-term storage at RT [8,9]. This low microstructure stability of the SPD materials processed at RT and lower temperatures did not occur in materials with high melting temperatures, such as Ti, Ni or Nb [10,11,12].

The creep behavior in SPD-processed pure metals showed that SPD-processed materials could exhibit an enhanced creep resistance compared to their standard coarse-grained (CG) states [13,14,15]. However, most of the published works investigating creep in the SPD-processes of pure metals at constant load were focused on FCC metals [14]. Only limited works are focused on the creep behavior of SPD hexagonal metals with a high T_m_ processed by SPD at RT or even cryogenic temperature [16,17,18]. 

At the present work, commercial purity Ti was rolled at elevated, room, and liquid nitrogen temperatures to establish the effect of temperature processing on a degree of microstructure refinement and describe the stability of the microstructure after static annealing and/or creep exposition. 

## 2. Materials and Methods

A commercial pure titanium VT1-0 with a total impurity content of about 0.3 wt.% (Fe, H, N, O, Al, and Si) was used as an experimental material (Table 1). From this material were prepared two types of ultrafine-grained states. The first one was prepared from a bar with a diameter of 30 mm. The bar was deformed by one step of helical rolling, one step of lengthwise rolling (both at 673 K), and, finally, again by helical rolling at ambient temperature to reach a final diameter of about 8 mm [19,20]. Thus, the deformed material corresponds to the imposed strain of about 1.5. This state will be referred to as state RT (room temperature state). This state was then additionally annealed for 3 h at 623 K to reduce internal stresses. 

The second state was processed by one step of lengthwise rolling. The 5-mm-thick sheet was cooled to close cryogenic temperature and then immediately rolled up to a thickness of 1 mm [21]. The equivalent strain imposed into the material was about 1.6. These states will be henceforth referred as state CT (cryo temperature state). The difference in SPD technology resulted in two different states with UFG microstructures. From both UFG states, a part of them was additionally annealed at temperatures of 823 K for 1 h. The creep specimens were cut from the as-pressed and annealed billets with gauge lengths parallel to the deformation direction. All flat tensile specimens had gauge lengths of 25 mm and a cross-section of 3 × 1 mm^2^. Constant load creep tests were conducted at different applied stresses with temperatures ranging from 648 to 723 K and under an argon atmosphere. All creep samples were run-up to the fracture. Microstructure investigations were conducted using a scanning microscope (SEM) Tescan Lyra 3 XMU FEG/SEM-FIB equipped with an EBSD unit and transmission electron microscope (TEM) Jeol 2100F operated at 200 kV. The subgrain and grain size were predominantly measured by the electron backscatter diffraction (EBSD) technique. The misorientation of θ = 15° between neighbouring areas was considered to distinguished subgrains surrounded by low-angle grain boundaries (LAGBs, θ < 15°) and grains surrounded by high-angle grain boundaries (HAGBs, θ ≥ 15°). The grain size in the CP state before the creep testing was determined from TEM micrographs due to the insufficient spatial resolution of the EBSD. The grain and subgrain size was determined using a standard line intercept method. 

## 3. Results

### 3.1. Microstructure of SPD-Processed Ti

**Figure 1:**
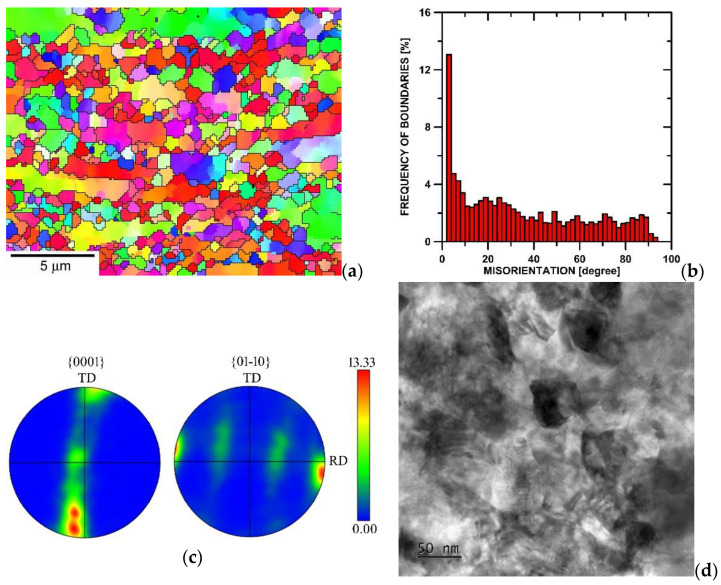
Microstructure of Ti (**a**) RT state, (**b**) grain boundary misorientation distribution for RT state, (**c**) pole figures for RT state, and (**d**) TEM micrograph of CT state.

Figure 1 shows the microstructure of Ti in the RT and CT states. The results demonstrate (Figure 1a) that the microstructure of the RT state is heterogeneous. The microstructure contains large grains elongated in the rolling direction and fine and nearly equiaxed grains. The measured grain size was about 0.6 µm and, in the microstructure, was detected as approximately 64% of the HAGBs (Figure 1b). The EBSD results also showed that the grains tend to form a strong fiber texture with <0–110> parallel to the rolling direction. The microstructure of the CT state was very fine and contained grains of about 50 nm in size.

### 3.2. Creep Resistance of Different SPD-Processed Titanium States

**Figure 2:**
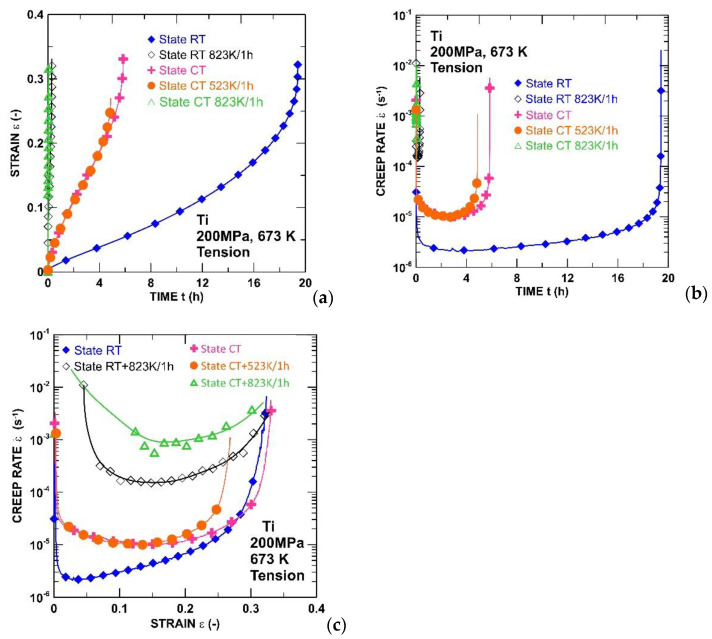
Creep dependence of (**a**) strain or (**b**) creep rate on time and (**c**) creep rate on strain for CP Ti processed by rolling methods.

Figure 2 shows the creep curves for the SPD processed CT and RT states and its post-SPD—annealing states for the uniaxial tensile creep test. All of these plots were acquired at a temperature of 673 K (which corresponds with ∼0.35 T_m_, where T_m_ is the melting temperature) and at an applied stress of 200 MPa. All of the creep tests were finished by fracturing the creep samples. As demonstrated in Figure 2, the RT state achieves an approximately five times better creep resistance than the CT state. Significant differences were found in the creep behavior of the as-received UFG states (RT, CT) when compared with their behavior after annealing (CTA, RTA). First, the standard ε vs. t curves appear to show that short-term isothermal annealing of the CT and RT states leads to a decrease of the creep fracture time, which is observed for both states. It was also shown that at a lower temperature of annealing (below 523 K), the test temperature led to an only slight decrease of the creep life, while a higher temperature of annealing (823 K) markedly degraded the creep strength for the same time of annealing. Similarly, the difference in the minimum creep rate between the as-received and aged states is about two or three orders of magnitude. Second, static annealing did not influence the final fracture elongation. Third, the as-received state and the annealing state differed considerably in the shapes of the creep curves. It should be noted that the creep curves in Figure 2a do not clearly indicate the individual stages of creep. However, these standard ε vs. t creep curves can be easily replotted in the form of the instantaneous creep rate $\dot{\varepsilon }$ versus time t, and/or in the form of the instantaneous creep rate $\dot{\varepsilon }$ versus strain ε (as shown in Figure 2b,c). Figure 2c gives us a better view of the individual stadium of creep. The dominant stadium in the CT state is represented by the primary creep. This creep represents approximately half of the creep time and deformation. A completely different course can be observed in the RT state, where, after a short strengthening in the form of the primary stage, there is an immediate transition to the tertiary stage and a subsequent fracture. The annealing states are rather close to the CT state, meaning that the initial strengthening-up to the deformation ∼0.1 is followed by a gradual softening, leading to the final fracture.

### 3.3. Determination of the Stress Exponent of Creep Rates for RT and CT States 

**Figure 3:**
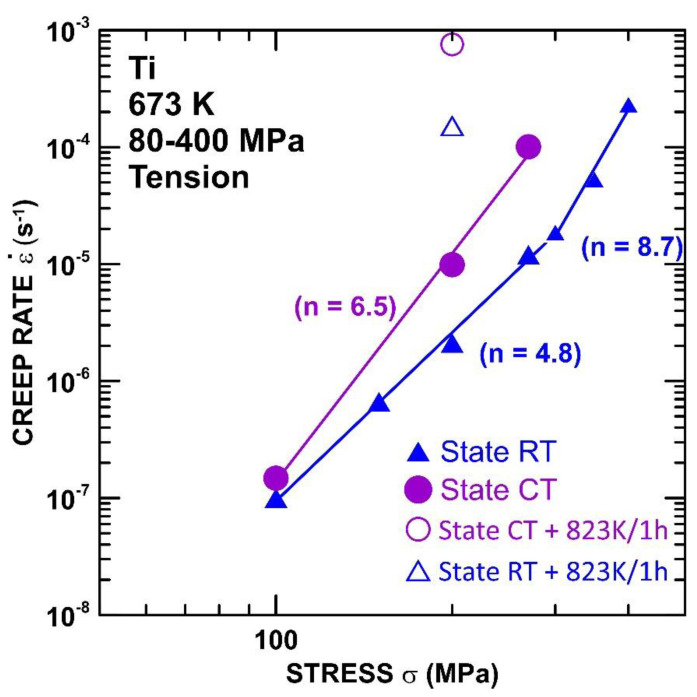
Stress dependences of creep rates determined at 673 K for RT and CT states.

In Figure 3, the minimum creep rate is plotted against the applied stress using a double logarithmic representation to attain the apparent stress exponents for the RT and CT states. The stress exponent *n* of the creep rate is determined from the following power law creep equation

(1) $$n={\left(\partial ln\;{\dot{\varepsilon }}_{m}/\partial ln\sigma \right)}_{T}$$

where ${\dot{\varepsilon }}_{m}$ is the minimum creep rate, and T is the absolute temperature. The results demonstrate that the ${\dot{\varepsilon }}_{m}$ measured at 673 K for the RT state is lower in comparison with the CT state. The stress dependences of the minimum creep rate (Figure 3) attained at 673 K demonstrate that the stress exponent *n* decreases from the value of *n*∼9 to the value of *n*∼5 at lower stresses. The CT state also shows similar values at lower stresses. Unfortunately, the results at higher stresses are not available due to a lack of samples. 

### 3.4. Monkman–Grant Relationships and Activation Energy of Creep

**Figure 4:**
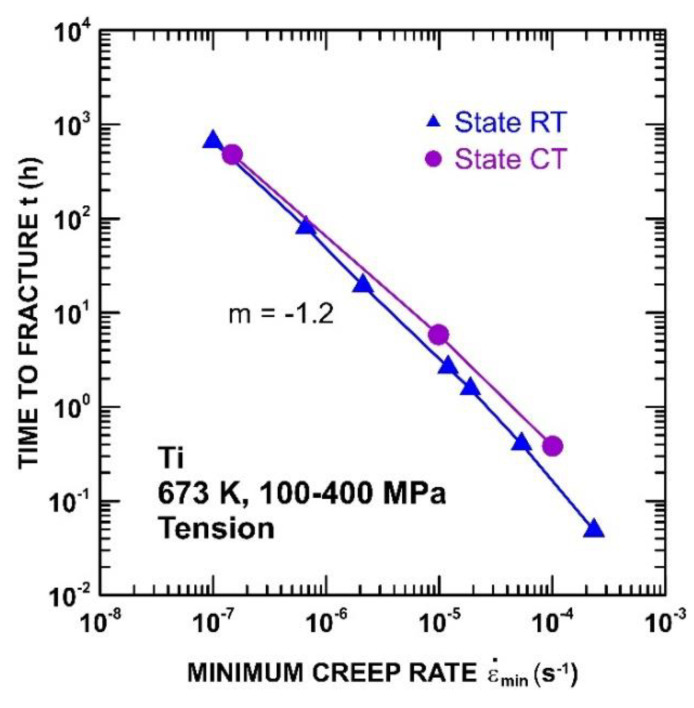
Monkman–Grant relationships determined for RT and CT states at 673 K.

The idea that the course of creep deformation and fracture are interconnected can be verified using the Monkman–Grant (MG) relationship [22,23], which shows that the minimum creep rate, ${\dot{\varepsilon }}_{m}$, is inversely proportional to the time of fracture, t_f_, according to the relation (${\dot{\varepsilon }}_{m}$) ω = CMG/t_f_, where ω is the exponent found in the literature about 1.0 [24,25] and CMG is constant. A graphical representation of the MG relationship for the creep data obtained at 673 K is shown in Figure 4. As shown in Figure 4, data for both the RT and CT states are well-fitted by this relationship with exponents very close to one, which suggests a relationship between the creep deformation and fracture processes.

**Figure 5:**
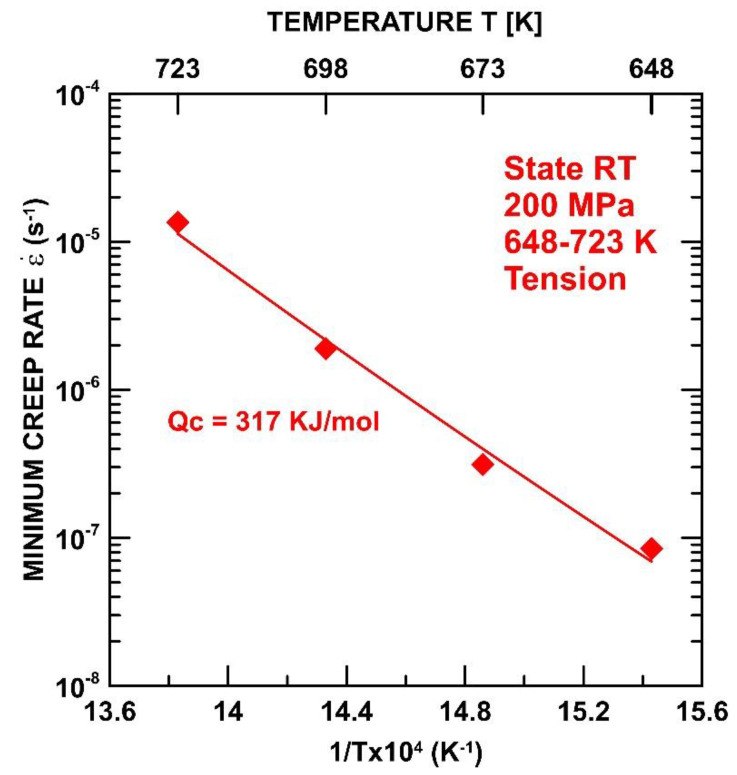
Activation energy for (steady-state) creep of RT state as a function of temperature.

The activation energy for creep Qc was determined experimentally from the line attained by the plotting dependence of the temperature at the minimum creep rate. Experimental data were achieved from temperature intervals from 648–723 K and under an applied stress of 200 MPa. The value of activation energy for the UFG-RT state was about 317 kJ/mol (Figure 5).

### 3.5. Microstructure Analysis of SPD-Processed Ti after Creep Exposure

**Figure 6:**
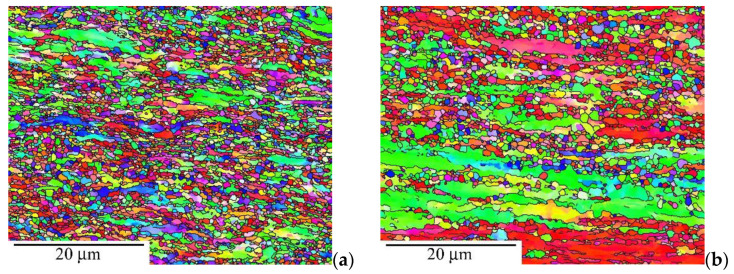

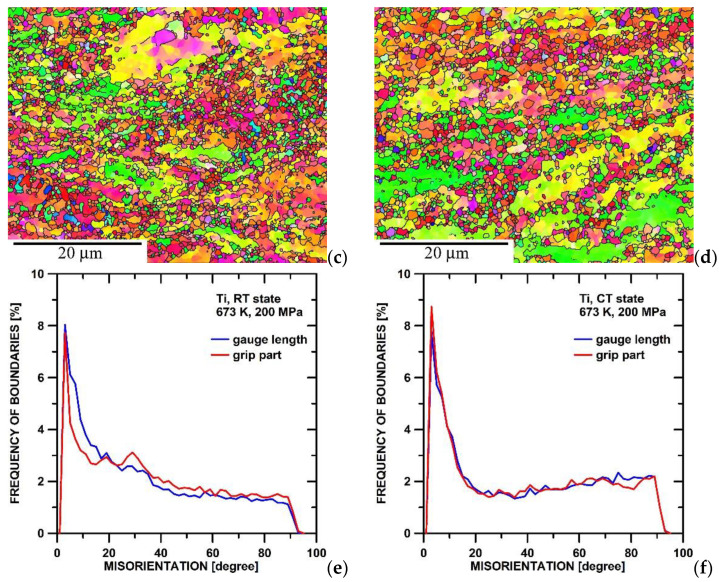
Microstructure of Ti after creep testing at 673 K and 200 MPa, (**a**) stress-free grip part of RT state, (**b**) gauge length of RT state, (**c**) grip part of CT state, (**d**) gauge length of CT state, (**e**) comparison of misorientation distributions of RT state after creep exposure, and (**f**) comparison of misorientation distributions of CT state after creep exposure.

Figure 6 shows the microstructures of the CT and RT states located in both the grip parts and gauge lengths of the specimens tested at 200 MPa and 673 K. The microstructures of both states after creep exposure are heterogeneous. The microstructure of the RT state (Figure 6a,b) after creep contains a mixture of large grains, significantly elongated to the rolling direction, and small, approximately equiaxed grains. One can see that the CT microstructure in the gauge length coarsens and contains long bands of large grains, which alternate with the areas of the fine grains (Figure 6c,d). 

The misorientation distributions (Figure 6e,f) show that both the microstructure of the RT and CT states contain a high number of boundaries in the gauge length and also in the grip part.

**Figure 7:**
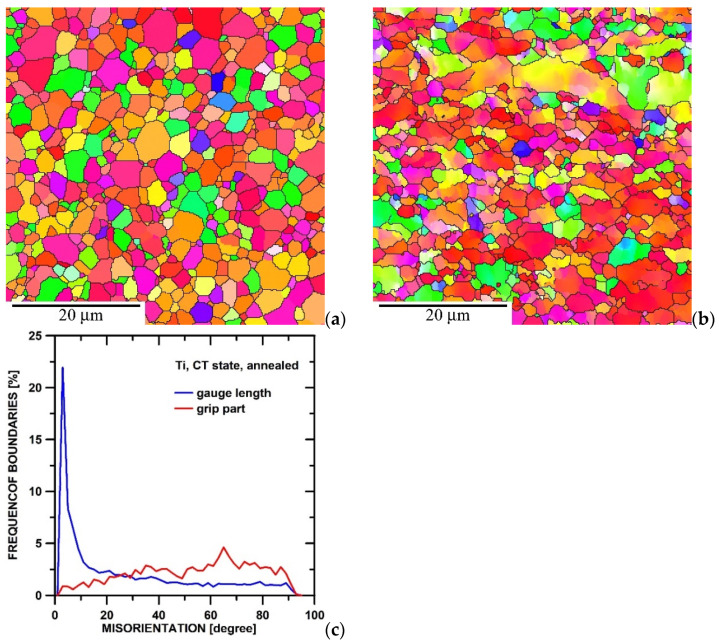
Microstructure of annealed CT state after creep testing at 200 MPa and 673 K: (**a**) grip part, (**b**) gauge length, and (**c**) misorientation distributions.

Figure 7 shows the microstructure of the annealed CT state (823 K/1 h) tested at 673 K and 200 MPa in the grip part and gauge length. One can see that the microstructure in the grip part includes large or small equiaxial grains (Figure 7a) that elongate in the gauge length during creep testing (Figure 7b). The comparison of the misorientation distributions demonstrates that the grip part of the statically annealed CT state predominates the HAGBs; however, tensile creep testing contributes to the formation of a high number of LAGBs in the gauge length (Figure 7c).

**Figure 8:**
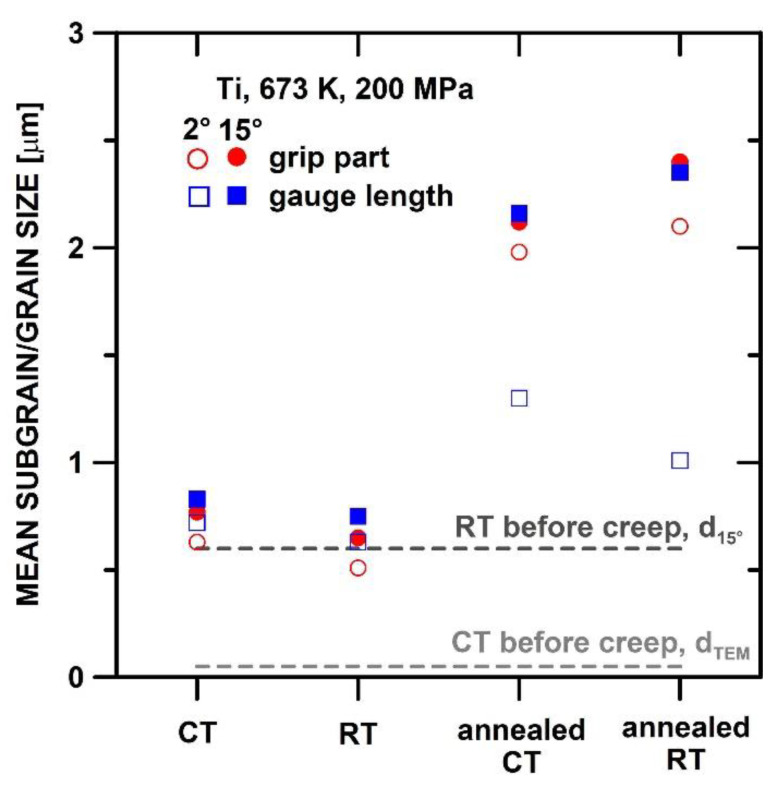
Comparison of grain and subgrain sizes in the grip parts and gauge lengths of CT and RT states tested at 673 K and 200 MPa.

Figure 8 shows the comparison of the mean grain and subgrain size measured in the gauge lengths and grip parts of the CT and RT states tested at 673 K and 200 MPa. It is seen that in the microstructure of the CT and RT states, the mean grain and subgrain size slightly coarsen during creep testing in comparison with the stress-free grip parts. 

The results also demonstrate that annealing at 823 K/1 h before creep testing leads to significant grain and subgrain coarsening in the CT and RT states. One can see that the mean grain size in the grip parts and gauge lengths of the annealed states is more or less similar. However, the mean subgrain size is reduced significantly during creep in the gauge lengths in comparison with the mean subgrain size in the grip parts.

## 4. Discussion

It is generally known that the application of severe plastic deformation (SPD) methods leads to the significant refinement of the grain size and formation of an ultrafine or even nanocrystalline microstructure [1]. The formation of an ultrafine-grained microstructure occurs by the gradual increase of the misorientation of the LAGBs, which are transformed into HAGBs. It was shown that the largest reduction of grain size usually occurs up to an equivalent strain of about four when the SPD-processed microstructure contains predominantly HAGBs [14,26,27]. In order to form a microstructure in pure Ti-containing grains finer than 100 nm, an equivalent strain, larger than 20, may be required [12,28,29,30,31]. However, in the present work, the equivalent strain imposed into the material was only up to two. For this reason, the standard deformation methods, such as rolling, are not as effective in grain size refinement compared to an HPT or ECAP. However, the results showed that it is not necessary to impose high values of an equivalent strain into the material in order to achieve a microstructure containing nanograins. Another important factor that affects grain size refinement is the temperature of the SPD deformation. The reduction of the SPD processing temperature to a cryogenic level makes conventional rolling an effective SPD technique, although the imposed equivalent strain is not too high.

The previous works [5,6,7] investigating the microstructure of SPD materials processed at a cryogenic temperature found that pure metals with a low melting temperature have a low microstructure stability, and the SPD-processed microstructure can recrystallize even at room temperature. The lower thermal stability of the microstructure deformed at a cryogenic temperature was also found in the present work. The current results (see Figure 1) showed that the CT-processed Ti achieved a finer grain size than the Ti rolled at room temperature. However, after creep exposure, the mean grain size of the CT-processed Ti was slightly larger than the grains in the RT-processed Ti. 

**Figure 9:**
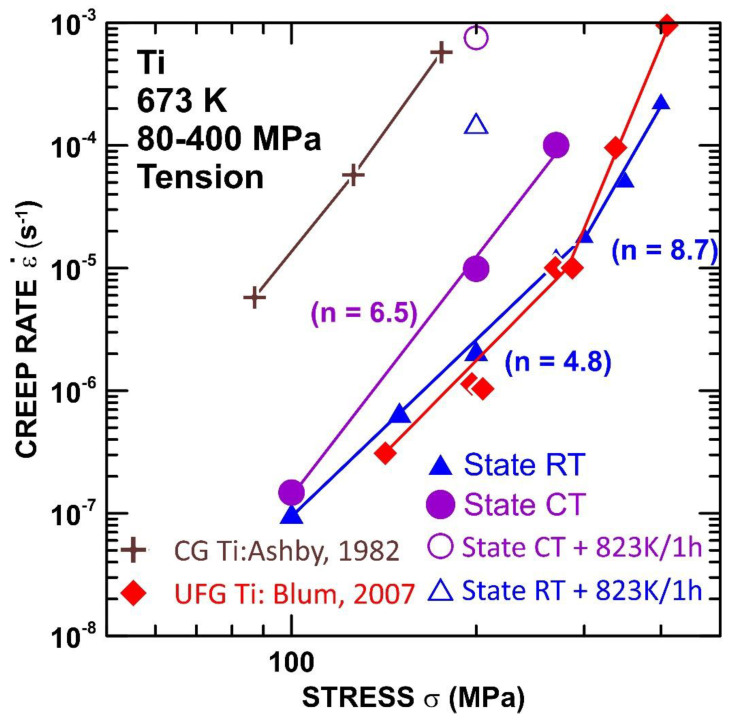
Comparison of the creep behaviour of titanium for different states of microstructure [32,33].

Figure 9 shows the comparison of our present creep results with the creep results published for CG Ti [32] and UFG Ti [33]. These results show that the annealing of SPD-processed Ti at 873 K/1 h led to the faster ${\dot{\varepsilon }}_{min}$, which approaches ${\dot{\varepsilon }}_{min}$ when determined for CG Ti with a grain size of 100 µm [32]. The microstructure results suggested that the increase of ${\dot{\varepsilon }}_{min}$ in the annealed CT and RT states could be related to the microstructure coarsening. 

The RT state exhibited a slightly finer subgrain size in the gauge length than the CT state, both in the initial and also in the state annealed at 873 K/1 h before the creep (Figure 8). However, these differences are not large enough to explain the significant differences in ${\dot{\varepsilon }}_{min}$ and time-to-fracture. Figure 9 shows that the ${\dot{\varepsilon }}_{min}$ determined for the RT state is more or less similar to the ${\dot{\varepsilon }}_{min}$ published for UFG commercial purity Ti [33]. The UFG Ti investigated in the work [33] was processed by eight ECAP (equal channel angular pressing) passes, with the initial temperature at 723 K; the temperature decreased with the following passes and reached 673 K at the eighth ECAP pass. We would like to point out that the RT state investigated in the present work was annealed at 623 K/3 h, after rolling, in order to relieve the internal stresses. Our results showed that the RT-processed state exhibited a short primary stage (Figure 2c). However, the CT state processed at a cryogenic temperature exhibited a long primary stage. For this reason, the occurrence of a short primary stage is associated with thermal heating during or after the application of the SPD. It is generally accepted that the short primary stage is related to a low density of free dislocations [34,35]. Thus, the creep results suggest that the CT state exhibits a higher density of free dislocations in comparison with the RT state. This can be attributed either to the recovery of the free dislocations during the annealing of the RT state before the creep or to the formation of some obstacles that restrict the movement of the dislocations. It can be suggested that the higher creep strength of the RT state in comparison with the CT state is influenced by impurities, which can form the precipitates or solute atmospheres around the dislocations and hinder their movement. Blum et al. [33] also suggested that UFG Ti exhibits dynamic strain aging effects attributed to the interaction of the dislocations with solute atmospheres. Zhang et al. [36] studied the effect of a small amount of solute on the strength and microstructural stability of HPT-processed Ni, and they found that the presence of a small amount of Ti in the solute solution caused an unexpected effect on the microstructure refinement, increase of the strength, and thermal stability. 

Valiev et al. [37] observed the effect of short-term annealing on the strength and ductility of UFG Ti processed at room temperature using HPT (high-pressure torsion). They revealed that annealing at 573 and 623 K for 10 min caused a significant increase in both the strength and ductility of UFG Ti without a visible change in grain size. It was suggested that the improvement of the mechanical properties in UFG Ti after short, low-temperature annealing could be associated with the ordering of the defect microstructure along the grain boundaries, leading to the enhancement of grain boundary sliding (GBS). However, the increased contribution of GBS rather leads to a decrease in the strength of the UFG materials [14], and it can only explain the higher ductility.

From the creep data reported in this study, it has been possible to extract activation energy values. The grain boundary sliding is controlled by grain boundary diffusion, and the value of the activation energy for the creep should be close to the activation energy for the boundary diffusion Q_b_ = 97 kJ/mol [32]. Previous works [13,14,15] investigating the creep behavior of SPD-processed materials showed that SDP-processed materials exhibit lower values of Q_c_ compared to their CG counterparts. However, in the present work, the value of Q_c_ ∼317 kJ/mol was evaluated, which is more than three times higher in value than Q_b_. The value of Q_c_ was determined at a stress of 200 MPa. Figure 3 shows that the stress dependences of the RT states are not interpolated by one straight line, which suggests that the stress dependence of the minimum creep rates cannot be predicted by a simple power equation with a constant value of the stress exponent *n*. In this case, the value of the stress exponent *n*, measured at a temperature of 673 K, increases with the increasing σ from a value of approximately ∼5 at the lower stresses (up to 300 MPa) to a higher value of ∼9 at the higher stresses. It is well known that a value of *n* = 1 corresponds to the diffusion creep while *n* = 3–8 is attributed to the dislocation (power-law) creep. The value of *n* found in this work suggests that the dominant mechanism is a power-law creep, which involves an intragranular dislocation movement, such as the dislocations’ glide and climb. The dislocation movement during the power-law creep is controlled by the lattice self-diffusion [38]. However, the value of Q_c_ is measured at higher than the value reported in the literature for the activation energy for self-diffusion [39] and the activation energy for the creep measured for CG Ti in the region of power-law [32]. The value of Q_c_ ∼ 317 kJ/mol determined in the present work approaches the value of Q_c_ ∼ 329 kJ/mol for the activation energy for an Al solute diffusion [39]. 

## 5. Conclusions

An important factor of SPD processing that affects grain size refinement is temperature. It was shown that Ti rolled at a cryogenic temperature achieved a finer grain size than Ti rolled at room temperature. However, the thermal stability of the microstructure deformed at a cryogenic temperature is not high, and the mean grain size measured after the creep exposure of the CT-processed Ti is slightly larger than the grain in the RT-processed Ti. 

The creep test revealed that the RT-processed Ti has a higher creep resistance in comparison with the CT-processed Ti. This effect can be influenced by impurities in the RT-processed state, which can form precipitates or solute atmospheres and effectively restrict the dislocation motion. The static annealing of the UFG states led to the degradation of the creep properties due to microstructure coarsening. It was found that under creep testing conditions, using the dominant operating mechanism, is a power-law creep, which involves intragranular dislocation movement, such as the glide and climb of the dislocations.

## Figures and Tables

**Table 1:** Chemical composition in wt.% of investigated states of titanium VT1-0.

Element	Al	Si	Cu	Fe	O	C	N	Ti
CT state	0.35	0.01	0.069	0.1	0.02	0.007	0.009	Bal.
RT state	0.01	0.002	-	0.12	0.143	0.004	0.003	Bal.

## Data Availability

Data sharing is not applicable to this article.
